# Automated application of low energy electron irradiation enables inactivation of pathogen- and cell-containing liquids in biomedical research and production facilities

**DOI:** 10.1038/s41598-020-69347-7

**Published:** 2020-07-30

**Authors:** Jasmin Fertey, Martin Thoma, Jana Beckmann, Lea Bayer, Julia Finkensieper, Susann Reißhauer, Beatrice Sarah Berneck, Leila Issmail, Jessy Schönfelder, Javier Portillo Casado, Andre Poremba, Frank-Holm Rögner, Bastian Standfest, Gustavo R. Makert, Lia Walcher, Ann-Kathrin Kistenmacher, Stephan Fricke, Thomas Grunwald, Sebastian Ulbert

**Affiliations:** 1 Fraunhofer Institute for Cell Therapy and Immunology IZI, Perlickstrasse 1, 04103 Leipzig, Germany; 2 Fraunhofer Institute for Manufacturing Engineering and Automation IPA, Nobelstrasse 12, 70569 Stuttgart, Germany; 3 Fraunhofer Institute for Organic Electronics, Electron Beam and Plasma Technology FEP, Winterbergstrasse 28, 01277 Dresden, Germany

**Keywords:** Engineering, Biomedical engineering, Biologics, Biotechnology, Applied immunology, Vaccines

## Abstract

Ionizing radiation is widely used to inactivate pathogens. It mainly acts by destroying nucleic acids but causes less damage to structural components like proteins. It is therefore highly suited for the sterilization of biological samples or the generation of inactivated vaccines. However, inactivation of viruses or bacteria requires relatively high doses and substantial amounts of radiation energy. Consequently, irradiation is restricted to shielded facilities—protecting personnel and the environment. We have previously shown that low energy electron irradiation (LEEI) has the same capacity to inactivate pathogens in liquids as current irradiation methods, but generates much less secondary X-ray radiation, which enables the use in normal laboratories by self-shielded irradiation equipment. Here, we present concepts for automated LEEI of liquids, in disposable bags or as a continuous process. As the electrons have a limited penetration depth, the liquid is transformed into a thin film. High concentrations of viruses (Influenza, Zika virus and Respiratory Syncytial Virus), bacteria (*E. coli, B. cereus*) and eukaryotic cells (NK-92 cell line) are efficiently inactivated by LEEI in a throughput suitable for various applications such as sterilization, vaccine manufacturing or cell therapy. Our results validate the premise that for pathogen and cell inactivation in liquids, LEEI represents a suitable and versatile irradiation method for standard biological research and production laboratories.

## Introduction

The inactivation of pathogens is a crucial step in many aspects of biological and medical research and production processes. Although several ways exist to inactivate viruses or bacteria by physical or chemical methods, the biological material is usually severely damaged or destroyed, which does not allow any down-stream applications. In contrast, few inactivation technologies leave the structural components largely intact, so that the material can be used e.g. as vaccines, in diagnostics or in therapeutic strategies^1–3^.

Ionizing radiation, such as gamma-, X-rays or high energy electrons, has been used for decades for the inactivation of pathogens, e.g. in sterilizing food or packaging^4–6^. It acts by treating the material with particles or photons of high energy, which leads to breaking of atomic bonds and the formation of radicals. Ionizing radiation usually acts faster than chemical treatment, and the inactivation of pathogens is predominantly based on the destruction of nucleic acids, whereas structural components such as proteins remain largely intact^7,8^. This effect might be based on the relatively large size of the DNA molecule in comparison to proteins. Particles will hit the DNA more often, leading to cell death or inability of DNA replication. Moreover, cells or viruses usually contain many identical copies of the same protein, and damaging of a few of them might cause only little effect on the overall cellular function. Consequently, irradiated viruses or bacteria are highly efficient as vaccines against a variety of human or animal infections, and the technology can be used to inactivate pathogens in various biological samples^9–11^. There are several advantages over chemical inactivation (currently the only way to produce inactivated vaccines), including the lack of toxic chemicals like formaldehyde, high reproducibility, high conservation of antigenic content and short reaction time^3,9,12–14^.

The inactivation of viruses and bacteria, however, requires high doses of rsubsequently inserted into the modadiation (in the kilogray range). Ionizing irradiation facilities therefore require a separated irradiation compartment surrounded by a combination of heavy lead shielding and/or thick concrete walls and cannot be placed inside or even close to biological production or research laboratories^15,16^. This drawback has so far prevented ionizing irradiation-based inactivation processes in many biological and medical applications. The only exception is ultraviolet (UV) light of short wavelength, also considered ionizing irradiation, which does not generate such secondary high-energy photons, but can cause substantial damage to the biological material, e.g. due to photoadducts, and generates less efficient vaccines compared to gamma-irradiation^17–19^.

As an alternative method, low energy electron irradiation (LEEI) uses accelerated electrons with energies between 150 and 300 kilo-electron-volts (keV) and generates only very limited amounts and lower energy of secondary photon radiation (Bremsstrahlung, X-rays). It therefore requires less radiation shielding (millimeters to centimeters, depending on the energy used) and can be used in normal laboratories. As low energy electrons only have a limited penetration depth, they have so far mainly been used for the sterilization of surfaces^20–22^. We have previously shown that LEEI can be used to inactivate pathogens in liquid solution, as long as the layer thickness is below 150 μm. The antigens of the irradiated viruses and bacteria have proven to be largely intact and elicited protective immune responses in animals^23–25^. However, these experiments were performed in a scale of maximally 200 μL, and to obtain the necessary low liquid height for irradiation, the suspension was covered by hand using a piece of foil^23^. Hence, despite providing a proof-of-principle, this approach was not suitable for implementing the use of LEEI beyond the research scale.

To overcome these limitations, we present key concepts for automated LEEI of liquids in various applications. Two modules were developed and constructed which transform the liquids into thin films, suitable to be penetrated by low energy electrons. Solutions containing pathogens and cells were inactivated and remained functional for diagnostic and therapeutic applications as well as vaccines.

## Materials and methods

### Low energy electron irradiation

The samples were irradiated in a custom-built electron beam device, equipped with an electron emitter of maximally 300 keV (type EBA 300/270/4, ebeam Technologies) (supplementary Fig. 1), and situated in a BSL2 laboratory at the Fraunhofer Institute for Cell Therapy and Immunology. The distance between electron exit window and substrate was 35 mm.

For all irradiation experiments the acceleration energy was set to 200 keV, except for Influenza A viruses in the disposable bag module, which were irradiated with 300 keV. Adjustments of irradiation doses were made by regulation of the beam current at a constant sample speed. To achieve the low doses for irradiation of mammalian cells, the electron flux was reduced mechanically by installation of a molybdenum slit diaphragm on the electron exit window with a slit clearance of 0.5 mm.

**Figure 1:**
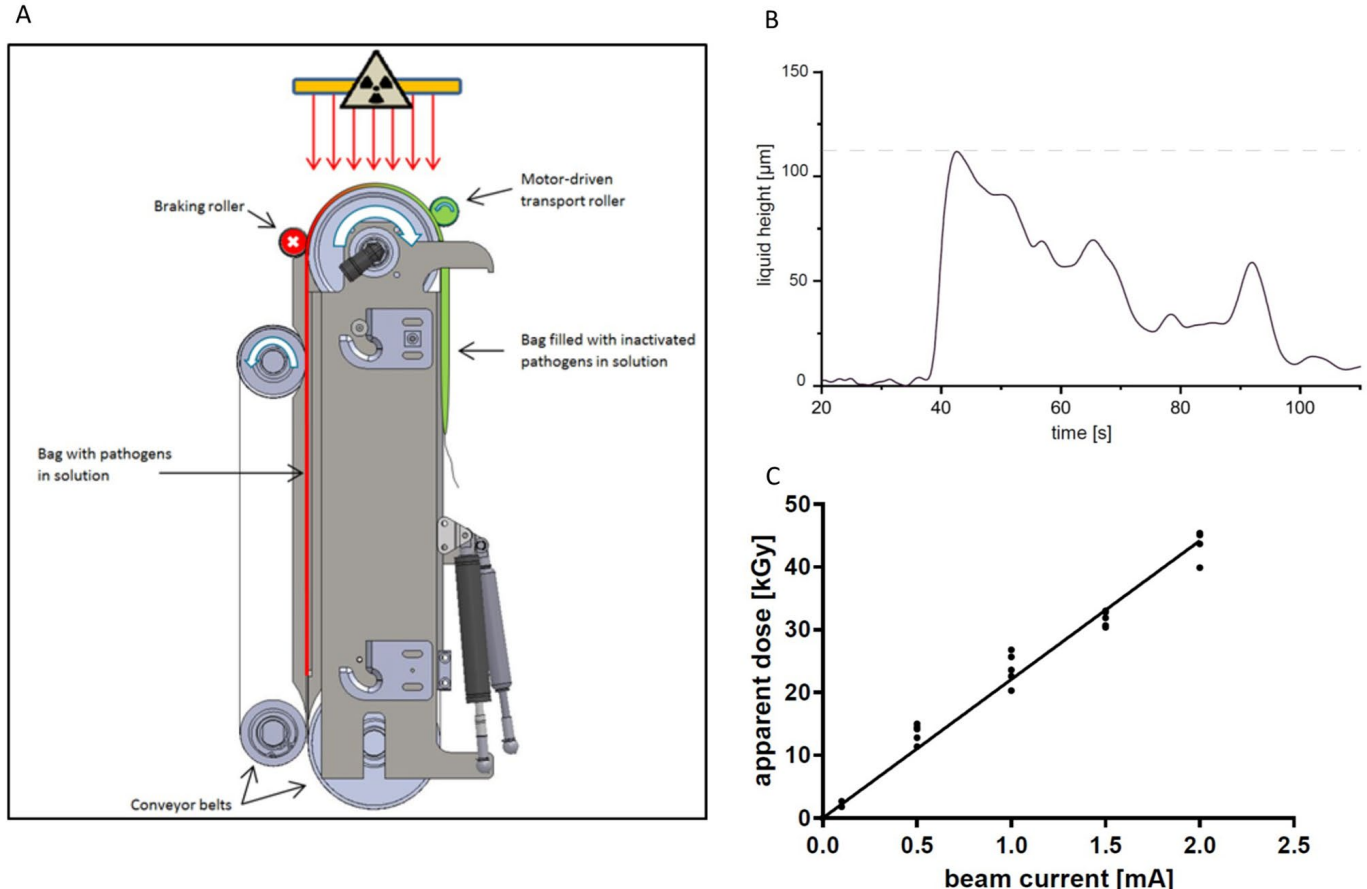
LEEI treatment of liquids filled in disposable bags. (**A**) Schematic drawing of the module for automated LEEI in disposable bags. The liquid is filled in disposable bags which are transported over conveyor belts and pass the irradiation window (radiation symbol). The bag is stretched using a braking roller that limits the bag height during processing. (**B**) Representative measurement of the bag height during the irradiation process. Measurement was conducted with PBS. The Y-axis represents the measured height [µm] of the bag over time (X-axis) until the bag is completely processed. The grey dashed line indicates the maximum height. (**C**) Apparent dose in kilo-gray (kGy) using a TTC-based liquid dosimeter system depending on beam current (mA) (n = 3). Each dot represents one single measurement. The line shows a linear regression (R^2^ = 0.974).

**Figure 2:**
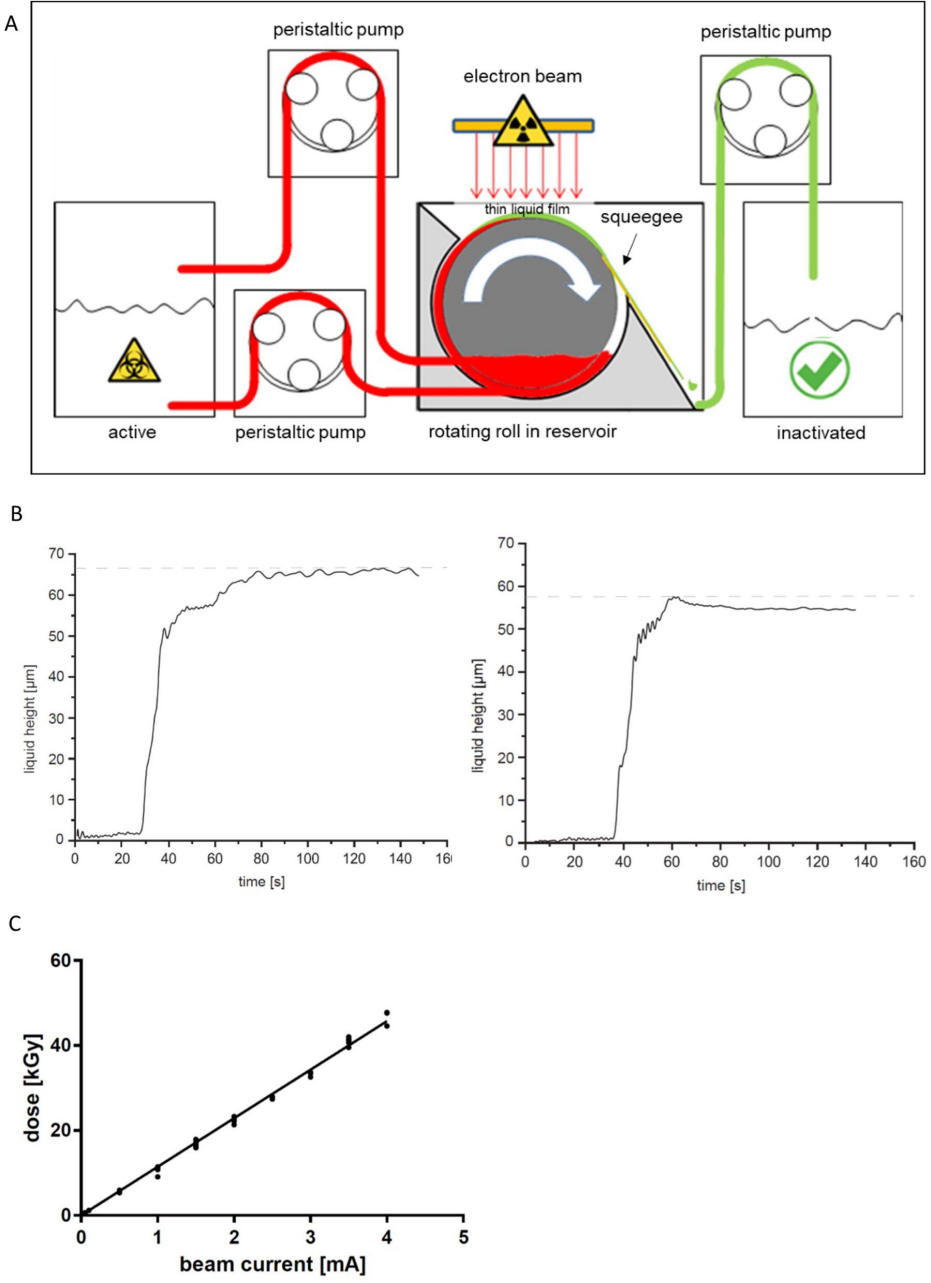
LEEI treatment of liquids in a continuous system. (**A**) Left: schematic drawing of the liquid roll module for automated LEEI. The liquid containing the pathogens (active) is pumped into a reservoir where a roll is rotating. The roll transports the liquid through the irradiation window (radiation symbol). After irradiation the inactivated solution is wiped off the roll by a squeegee and collected in a separate container (inactivated). (**B**) Measurement of the height of the liquid film during the irradiation process. Left: Measurement was performed with PBS and 20% Glycerol (w/v). Right: Measurement was performed with PBS and 10% Sucrose (w/v). Each curve is representative for one out of three independent measurements. The Y-axis represents the measured height of the liquid over time (depicted on X-axis). The grey dashed line indicates the maximum height. (**C**) Measured doses in kGy depending on beam current using calibrated foil dosimeters placed directly on the roll (n ≥ 3). Each dot represents one single measurement. The line shows a linear regression (R^2^ = 0.995).

The two modules for automated LEEI were constructed as research prototypes – one using disposable bags (Fig. 1A and supplementary Fig. 1B) and the other employing a continuous process (Fig. 2A and supplementary Fig. 1C) and will be described in detail elsewhere (MT and BS, manuscript in preparation). The modules can be cooled during processing, and all LEEI-experiments were performed at 4 °C, except for natural killer cells (NK-92), which were irradiated at room temperature. The velocity for sample transport was fixed according to the given parameters of each module (disposable bag module: 5 mm/s bag transportation velocity; continuous system: 120 mm/s circumferential speed of the roll). For LEEI treatment using the bag system, disposable polyethylenterephthalat bags (39 cm length/10 cm width) were produced in-house (PET-O/PE 12/50 (Coveris), thickness 62 µm, density 1.017 g/cm^3^) and filled with up to 20 ml of the liquid, which were sealed using a sealing machine (D 545 AH-2, Kopp Verpackungssysteme) and subsequently inserted into the module, followed by initiating the irradiation procedure. In short, the bag with the liquid is tensioned and thus flattened before it is fed into the LEEI zone, such that the liquid film inside the bag does not exceed a maximum layer thickness of 110 µm. For LEEI treatment in the continuous system, glass bottles (Schott, Germany) were filled with the liquid, which were then transferred into the module. The liquid is pumped into a reservoir where a stainless-steel roll is wetted by surface tension and transports the liquid through the LEEI zone. Fresh tubes were used for collecting the irradiated sample. The product-contacting components of the modules are designed to be exchangeable cassettes and can be sterilized, e.g. via autoclaving. After irradiation, bags or tubes were taken out of the device, and the samples were prepared for further experiments or storage. To ensure sterility, the bags were handled in a safety cabinet and sealed for transport and irradiation as described above. After LEEI, the bags were opened using sterile scissors, and the liquid was removed by pipetting and transferred to sterile tubes. Controls underwent the same handling procedures, except that no LEEI was applied.

### Measurement of liquid height

A system based on the diffraction of coherent light at the single slit was developed to measure the liquid height during LEEI irradiation. In brief, a razor blade was mounted at a small distance above the roll, creating a slit between the razor blade and the roll (supplementary Fig. 2). When liquid (either inside a bag or directly on the roll) is processed, the razor blade makes up the top of the diffraction slit, while the liquid or the bag surface make up the bottom part, in the continuous module and the bag-based module, respectively. Light from a laser with a wavelength of 685 nm is diffracted at the slit and the resulting interference pattern is projected directly onto the sensor of a camera (Basler acA4112-30 um). From the distances of the interference minima (depending on the changing slit width), the distance from the slit to the camera sensor (210 mm) and the pitch of the pixels (3.45 μm × 3.45 μm), the slit width and thus the height change of the liquid were calculated. The calculations from the diffraction measurements were carried out with an in-house, self-written software (supplementary note 1). For measurements in the continuous system, different buffer compositions (PBS with either 20% glycerol or 10% sucrose) were used, as viscosity leads to an increase of liquid height. The layer thickness inside the bags was measured with buffer + 20% glycerol only, which had the highest viscosity of the analyzed samples in the continuous system. Thus, only the maximum thickness was investigated. Currently this method for determination of the liquid height is adapted to allow a continuous process monitoring during irradiation.

### Dose measurements

At present, no standard liquid dosimeter is available for LEEI. In order to measure the irradiation dose in the disposable bag system, a liquid dosimeter system was established based on 2,3,5-triphenyltetrazolium chloride (TTC) 0,2% (w/v) in water. The chemical forms red colored formazan upon treatment with ionizing radiation^26^, which was quantified by spectrometric analysis at a wavelength of 485 nm (Tecan infinite M200, Tecan Group Ltd.). For the measurements 60 µl of the TTC reagent were pipetted into wells of a 96-well plate (Greiner). The TTC response to an applied mean dose value of LEEI at room temperature was previously correlated to a calibrated radiochromic dosimeter film (Risø B3 dosimeter, Risø High Dose Reference Laboratory, Denmark) as a reference in the dose range of 6.5 to 38 kilogray (kGy). Calibration was carried out in a petri dish system using a foil of oriented polypropylene (OPP) to generate a thin liquid layer (approx. 85 μm) of TTC irradiated with a very low dose gradient of 13%^23^. For the irradiation with the slit diaphragm, a dose reduction of 99% was measured. Therefore, for the calculation of low doses a factor of 0.01 was applied. The measured dose reflects a mean dose value for the entire liquid solution within the bag. Validity of dose measurements at 4 °C is given up to a dose of 33 kGy with an estimated uncertainty range of 23.6%.

Due to its lower measurement uncertainty of 10.6%, absorbed dose measurements in the continuous LEEI system were carried out by fixing a calibrated radiochromic dosimeter film (Risø B3 dosimeter, Risø High Dose Reference Laboratory, Denmark) to the middle of the roll and performing one single rotation through the irradiation area at beam currents between 0.5 – 4 mA. At lower beam currents of 0.1 mA and 0.05 mA, the dosimeter had to pass the irradiation area 20 or 40 times, respectively, to achieve evaluable dose values within its calibration range. The color change of the dosimeter was quantified at a wavelength of 554 nm (RisøScan-System, Risø High Dose Reference Laboratory).

The film dosimeter represents the maximum dose at the surface independent from the thickness of the liquid film^27^. The critical parameter for determining the dose applied to the liquid surface is the velocity of the liquid, which in the present roll-device is assumed to be the same as the roll speed.

### Cultivation of bacteria

Cultivation of *E.coli* (DH5alpha, ThermoFischer Scientific, Germany) has been previously described^23^. Irradiation was carried out in PBS. *B. cereus* (DSM-31 synonym: ATCC 14579) was obtained from the Deutsche Sammlung von Mikroorganismen und Zellkulturen (DSMZ, Germany) and grown over night in Nutrient Broth at 30 °C and rotation at 160 rpm. Sporulation was induced on the following day as previously described^28^ with minor modifications. In brief, the overnight culture was harvested by centrifugation (4,600 rpm for 10 min) and resuspended in fresh nutrient broth containing 0.01 mM MnCl_2_, 0.14 mM CaCl_2_, 0.20 mM MgCl_2_. Spores were harvested after 7 days by centrifugation (4,600 rpm for 10 min) and washed three times in sterile H_2_O. Sporulation was verified microscopically. Irradiation was carried out in sterile H_2_O.

To investigate the inactivation efficiency, colony-forming units were determined by serially diluting the irradiated and control samples in growth medium and plating on LB- (*E. coli*) or Nutrient Broth (*B. cereus*) agar plates. In parallel, 1 ml of the treated bacterial suspension was added to 4 ml of growth medium and incubated for 48–96 h at 160 rpm and at 37 °C or 30 °C respectively. When no growth was visible during that time, the sample was considered as inactivated.

### Cell culture

Cell lines were obtained from the DSMZ. K562 cells were cultured in RPMI1640 (ThermoFisher Scientific, Germany), VeroE6 cells were cultured in DMEM (ThermoFisher Scientific, Germany) medium, both supplemented with 10% heat inactivated FBS (Gibco). For NK-92 cells, alpha-MEM (with ribo- and deoxyribonucleosides, Sigma-Aldrich, Germany) medium supplemented with 10% heat inactivated FBS (ThermoFisher Scientific, Germany), 10% heat inactivated horse serum (Sigma-Aldrich), 2 mM l-glutamine (Sigma-Aldrich) and 100 IU/ml interleukin-2 (Peprotech, USA) was used. Cell number and viability were determined using CASY cell counter and analyzer (Schärfe System GmbH). All cell lines were maintained at 37 °C with 5% CO_2_. Hep2 cells were used for RSV in vitro assays and MDCK cells for Influenza A (H3N8) propagation, their cultivation have been previously described elsewhere^23,24^.

For cell irradiation, 10^7^ NK-92 cells in a volume of 20 ml culture medium per irradiation bag were used. After irradiation, the cell number was determined and adjusted to 0.3–0.5 × 10^6^ viable cells/ml in fresh medium. The cells were analyzed for a period of up to 13 days after irradiation, during which the cell concentration was adjusted at least every 4 days as described above.

### Virus cultivation

Cultivation and purification of respiratory syncytial virus (RSV) (strain Long) and Influenza A/H3N8 (strain A/equine/Miami/2/63) have been described^23,24^. Zika virus (ZIKV) strain PD1 (obtained from University of Padova)^29^ was propagated in Vero E6 cells. Inactivation was tested by serially inoculating 100 µl per well of the irradiated and control samples to 6 well cell culture plates, seeded with the respective cell line. Testing was performed in duplicates, and development of a cytopathic effect (CPE) was monitored regularly. Inactivation was confirmed by serial passage of the supernatant to fresh cells. When no CPE was visible after passage, the sample was considered as inactivated.

### Determination of antigen conservation

Enzyme-linked Immunosorbent assays (ELISAs) for antigen conservation of *E.coli,* Influenza A and RSV were performed as previously described^23,24^. A human serum positive for ZIKV, and a negative serum were obtained from Padova University (Italy). Ethical approval was obtained from the Padova University Hospital Ethics Committee, with written informed consent from the patients. Rabbit sera from animals immunized with *B. cereus* (ATCC 14579) were obtained from CDC (USA). Hemagglutination assays for Influenza A were performed as previously described^23^. Analysis of CD56 integrity on irradiated NK-92 cells was performed by flow cytometry with a FACS Canto II flow cytometer (BD Biosciences). In brief, following blocking (Human BD FC Block, BD Biosciences, USA), 2 µL of CD56 antibody (PerCP-Cy5.5 mouse anti-human CD56 IgG1, κ, BD Biosciences, USA) were incubated with 1 × 10^6^ NK-92 cells for 20 min at 4 °C. Non-specific staining was evaluated with the isotype control PerCP-Cy5.5 mAb (PerCP-Cy5.5 Mouse IgG1 κ Isotype Control, BD Biosciences). Compensation was performed with UltraCom eBeads (ThermoFisher Scientific, Germany) and the absolute number of cells was determined using Precision Count Beads (BioLegend, USA). The mean fluorescence intensity (MFI) of the samples was calculated as described^30^. Details of the gating strategy are shown in supplementary Fig. 4 and Table 2.

Cell-mediated cytotoxicity was assessed in a standard 4 h chromium-release-assay. K562 target cells (3 × 10^5^ cells) were incubated with 25 µCi Chromium-51 radionuclide (Hartmann Analytic, Germany) for 1 h at 37 °C and 5% CO_2_. After labeling and washing, cells were co-incubated with NK-92 effector cells at an effector to target-ratio of 5:1 for 4 h. In addition, cells were also incubated with medium (spontaneous release) and 1% Triton-X100 (maximum release). 50 µl of supernatant were harvested and added to 150 µl of scintillation cocktail (Optiphase HiSafe, Perkin Elmer, Germany). Scintillation counts were acquired for one minute per well (Perkin Elmer MicroBeta Trilux 1450 LSC and Luminescence Counter). Specific lysis in percent was calculated as: Specific lysis = [(test release – spontaneous release)/(maximum release – spontaneous release)] * 100.

### RSV immunization and challenge

Female BALB/c mice (6–8 weeks old) were obtained from Charles River (Germany). Five mice per group were kept in a specific pathogen-free environment in isolated ventilated cages. All animal experiments were carried out in accordance with the EU Directive 2010/63/EU for animal experiments and were approved by local authorities (No.: TVV 07/15; DD24-5131/331/9).

50 µl LEEI-inactivated RSV containing 1.25 × 10^6^ TCID50 was mixed with 50 µl 2% Alhydrogel (Brenntag Nordic A/SSS, Denmark), per dose. Groups of mice were vaccinated twice in a 4-week interval by administration of 50 µl into the hind leg muscles. Control mice were not immunized. Blood samples were collected one week before immunization (pre-immune), three weeks after the first (prime) and four weeks after the second (boost) immunization. Analysis of RSV-binding antibodies by ELISA and RSV-neutralization tests were performed as previously described^24,31^. Four weeks after the boost, the mice were challenged with 30 µl PBS containing 1.4 × 10^6^ TCID50 RSV per animal after short inhalative isoflurane anesthesia. 5 days after infection, mice were sacrificed via isoflurane pre-anesthesia, followed by cervical dislocation. The viral load in the lungs was quantified by isolation of viral RNA and subsequent RT-qPCR as described previously^24^.

### DNA-detection of *B. cereus*

PCR was performed as previously described^32^, using the primer set specifically targeting a region in the 16S rDNA of the *B. cereus* group. Detection of the amplification product was performed using SYBRgreen and a LC 480-Cycler (Roche).

### Statistical analysis

GraphPad Prism 6.0.7 was used to perform statistical analysis using either one-way ANOVA, followed by Dunnet`s multiple comparisons test or T-test (unpaired, two-tailed). Probabilities lower than 0.05 were considered significant, level of significance is indicated by asterisk (*p ≤ 0.05; **p ≤ 0.01; ***p ≤ 0.001; ****p < 0.0001). In all figures shown, “n” indicates independent experiments, analyzed in technical replicates, if not stated otherwise.

## Results

As LEEI has a low penetration depth, we developed two separate strategies for the transformation of liquids into fluid films, which are thin enough to be completely penetrated by the electron beam while automatically moving through the irradiation area. In the first concept, the liquid is contained in a sealed disposable bag, whereas the second method enables continuous inactivation up to a multi-liter scale. Based on our previous work with small-scale irradiation, the thickness of the liquid film needs to be below 150 μm for efficient LEEI with 200–300 keV acceleration energy^23^. For each of the two strategies, a distinct module was constructed and subsequently inserted into the irradiation chamber containing an electron emitter (supplementary Fig. 1).

### Automated LEEI in disposable bags and in a continuous system

In the module for processing sealed bags, no mechanical components come into contact with the product. The bag (containing up to 20 ml of liquid) is placed between two conveyor belts (Fig. 1A) and is pulled over the conveyor´s top roller by a motor-driven transport roller. The liquid inside the bag is held back by a braking roller until the pressure is high enough to press it through the irradiation area below the electron source with approximately constant height and velocity. Subsequently, the liquid flows into the empty part of the bag that has already been irradiated.

The thickness of the liquid film (including the two layers of the bag material on top of and below the fluid) was determined with a laser interference measurement setup, as described in the methods section (supplemental Fig. 2). The maximum height was found to be 110 μm (Fig. 1B). The average processing time of the bags was 100 s per bag. To determine the applied irradiation dose, a liquid dosimeter system based on the chemical compound 2,3,5-triphenyltetrazolium chloride (TTC) was used. Due to the linear correlation between beam current and apparent dose, TTC measurement allowed an irradiation dose estimation as a mean value of the entire bag content with an uncertainty budget of 23.6% (Fig. 1C).

The second method of automated LEEI enables continuous processing within a completely closed system. The functional principle of the continuous process is shown in Fig. 2A: a roll is immersed in a reservoir of the liquid to be irradiated. The surface is continuously wetted by the rotation of the roll, and the liquid is thereby transported through the irradiation area, wiped off afterwards by a squeegee and transferred into a sterile container. Similar to the disposable bag-method, the thickness of the liquid film under LEEI was determined with the laser interference measurement setup (supplemental Fig. 2). Different buffer compositions (PBS with either 20% glycerol or 10% sucrose) were used, as viscosity was expected to have an impact on the liquid height. Indeed, buffer-dependent variations in the film thickness were observed. More viscous solutions resulted in thicker layers than pure cell culture supernatant (data not shown). However, for all buffer conditions tested, the values were well below 100 μm and therefore appeared suitable for full penetration by the low-energy electrons (Fig. 2B). As expected, the buffer composition also had an influence on the throughput, which was lower for cell culture supernatant as compared to more viscous liquids containing 10% sucrose or 20% glycerol, ranging from 27.9 (± 6.9) to 60.9 (± 8.7) ml/min (supplementary Table 1). To determine the applied maximum irradiation dose to the surface of the liquid, a commercially available, calibrated film-dosimetry with an uncertainty budget of 10.6% was used which revealed a linear and reproducible correlation between beam current and dose (Fig. 2C).

### Pathogen inactivation

**Figure 3:**
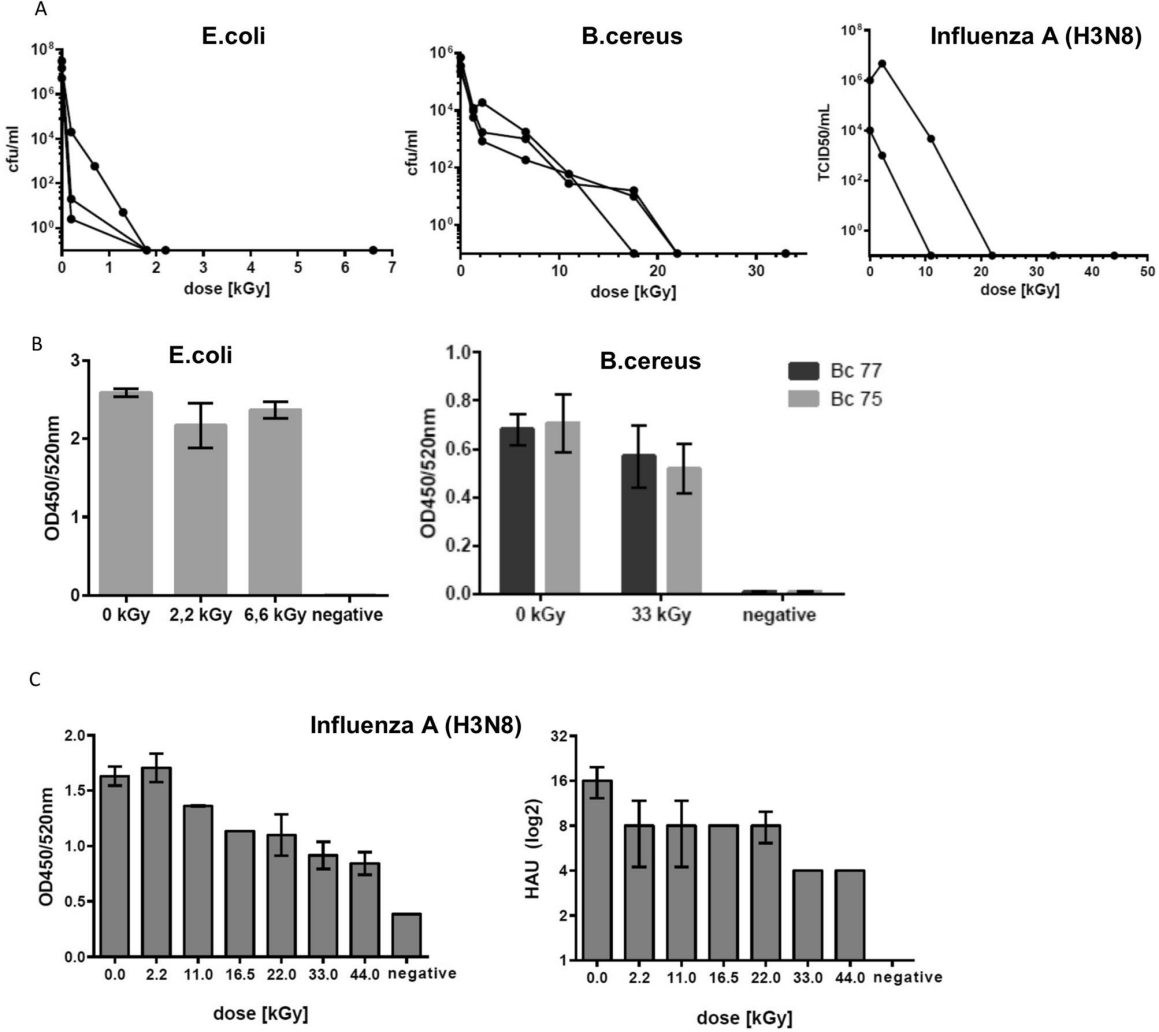
Different pathogens filled in disposable bags are efficiently inactivated by LEEI in the disposable bag module. (**A**) Inactivation curves: solutions containing purified *B. cereus* spores (n = 3), *E. coli* (n = 3), or Influenza A (H3N8) viruses (n = 2) were subjected to increasing doses of LEEI, and bacterial colonies or viral titers, respectively, were determined. (**B**) Antigenicity of inactivated bacterial samples: *B. cereus* spores (n = 3) and *E. coli* (irradiated with the indicated doses) (n = 3) were coated on microtiter plates and were analyzed in ELISA measurements using polyclonal antibodies. The antigenic content of the samples is indicated by the OD-signal obtained. (**C**) LEEI treated Influenza A (H3N8) containing cell culture supernatant using the indicated doses was analyzed by ELISA (left) and hemagglutination assays (right). Data from two and three independent experiments are shown, respectively, standard errors are indicated.

Next, we validated the functionality of automated LEEI in the disposable bag module for the inactivation of pathogens and investigated *Bacillus cereus*, a spore-forming gram-positive bacterium and the gram-negative *Escherichia coli.* Spores and *E. coli* cells were prepared, filled into the bags and subjected to LEEI. The inactivation of bacteria was dose dependent and application of 33 kGy resulted in inactivation of a *B. cereus* spore suspension with a mean titer of 4.33 × 10^6^ (± 2.5 × 10^6^) cfu/ml. For *E. coli* a dose of 2.2 kGy was sufficient to inactivate suspensions with a mean titer of 1.67 × 10^7^ (± 1.23 × 10^7^) cfu/ml (Fig. 3A). Inactivation curves were also established for a viral pathogen. Cell culture supernatants containing Influenza A viruses (mean titer of 5 × 10^5^ (± 7 × 10^5^) TCID50/ml) were inactivated after application of 22 kGy (Fig. 3A).

In order to analyze the conservation of structural components after irradiation, ELISAs with polyclonal sera were performed. For *B. cereus* spores, the signal intensity ranged between 73.3% (± 23%) and 83.5% (± 19.7%) of the non-irradiated sample depending on the serum that was used. Almost no difference between inactivated and non-inactivated *E. coli* samples was observed (83.7% (± 13.2%) and 91.4% (± 4.5%)), suggesting that antigenic structures were maintained after LEEI treatment (Fig. 3B). For Influenza A, ELISA-measurements demonstrated antigen-conservation of 67.3% (± 16.9%) at 22 kGy, the irradiation dose leading to complete inactivation. Hemagglutination activity of the same sample was reduced twofold (Fig. 3C).

While LEEI leads to the fragmentation of nucleic acids, DNA fragments might still be long enough to serve as template for diagnostic PCRs. We therefore tested whether *B. cereus* DNA in the inactivated samples could still be detected by PCR with an amplicon length of 288 bp. Quantitative Real-Time PCR was performed in three independent experiments and showed clear signals using *B. cereus*-specific primers, which were comparable between non-irradiated (0 kGy) and inactivated samples (33 kGy) with mean Ct-values of 14.83 (± 2.22) and 16.89 (± 2.68), respectively.

**Figure 4:**
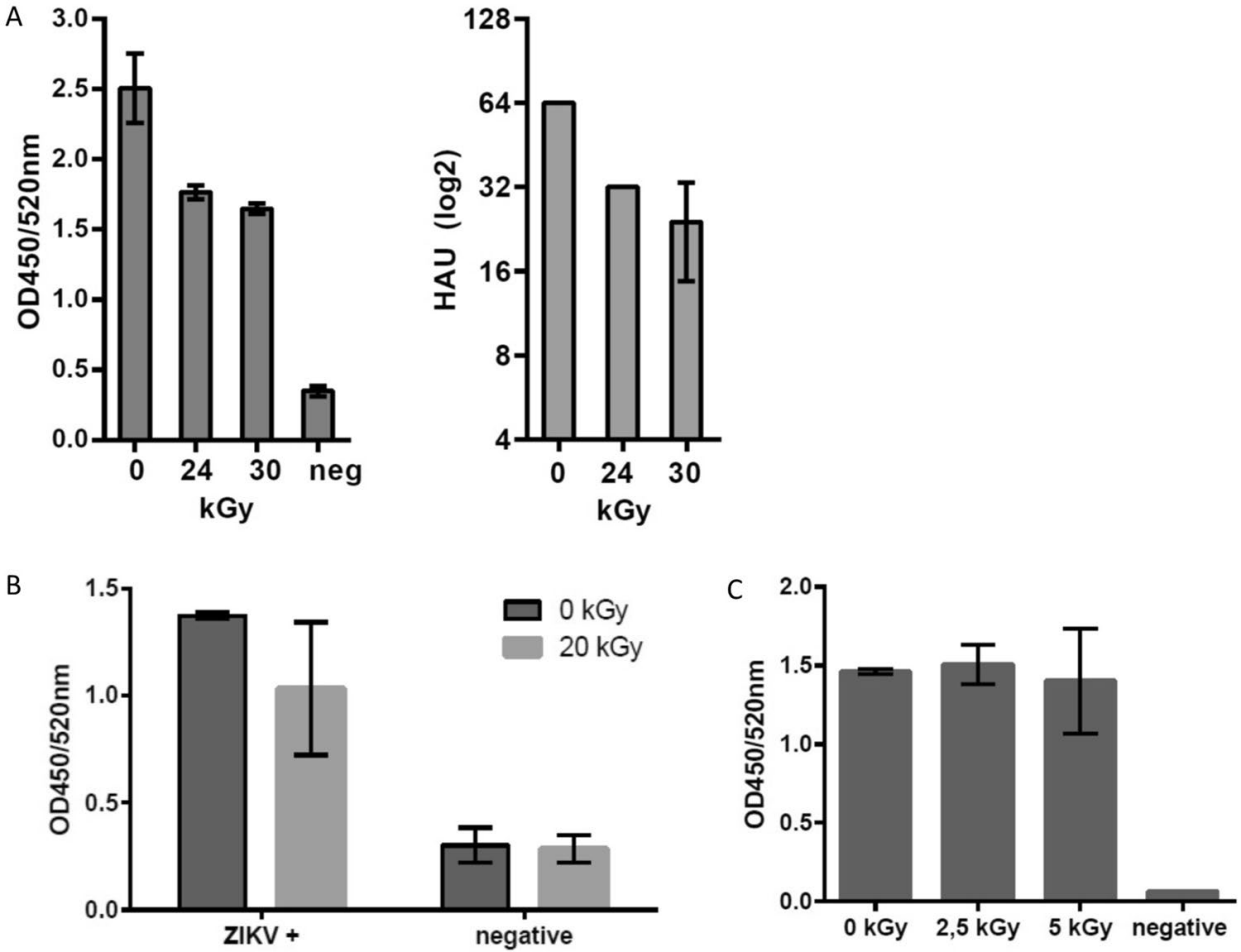
Viruses and Gram-negative bacteria are efficiently inactivated using continuous LEEI processing. (**A**) Influenza A (H3N8) containing cell culture supernatant was irradiated using the indicated doses. Inactivated samples (24 kGy and 30 kGy) were coated on ELISA plates for analysis with a polyclonal serum from an influenza-infected pig (left) and also tested in hemagglutination assays (right). Non-irradiated samples (0 kGy) served as positive control, cell culture supernatant from mock infected cells served as negative control (neg.). Data of three independent experiments are shown, standard errors are indicated. (**B**) Buffer containing purified ZIKV was irradiated with 20 kGy. After inactivation samples were coated on ELISA plates and analyzed using a Zika positive patient serum. Patient serum that was negative for Flaviviruses served as negative control. Data from three independent experiments are shown, standard errors are indicated. (**C**) *Escherichia coli* cells were irradiated in PBS + 20% Glycerol (w/v) using the indicated doses. Inactivated samples (2.5 kGy and 5 kGy) were coated on ELISA plates and analyzed with a polyclonal antibody targeting the O and K group antigens. Non-irradiated samples (0 kGy) served as control. Data from three independent experiments are shown, standard errors are indicated.

The inactivation of pathogens was also tested in the continuous system. Similar to the processing in bags, a dose of 2.5 kGy was sufficient to inactivate *E. coli* cells with a concentration of 2 × 10^7^ (± 1.3 × 10^7^) cfu/ml. We further analyzed the throughput of the continuous system. In two independent experiments, 800 ml of *E. coli* suspension (diluted in PBS + 20% Glycerol) were processed and inactivated within 15 min. In addition, virus suspensions of Influenza A, ZIKV and RSV were successfully inactivated (Table 1). Similar to the disposable bag system, the structural components of *E. coli*, Influenza A and ZIKV were analyzed by ELISAs with polyclonal antibodies. Influenza A antigenicity in the inactivated sample (24 kGy) was 70.4% (± 1.6%) as compared to the untreated material, and hemagglutination activity was reduced by a factor of two (Fig. 4A). A human serum positive for Zika virus was used to investigate antigenicity of the inactivated ZIKV (20 kGy). The serum displayed slightly reduced signals in the irradiated sample compared to the non-irradiated control (0 kGy). Only background signals were observed for both samples with Flavivirus negative sera (Fig. 4B). Antigenicity of inactivated *E. coli* (2.5 kGy) was not altered upon LEEI (Fig. 4C).

**Table 1:** Overview of pathogens that were successfully inactivated using the continuous LEEI module.

Pathogen	Concentration	Dose for complete inactivation (kGy)
Influenza A (H3N8)	1 × 10^6^ TCID50/ml	24
ZIKV	5 × 10^6^ TCID50/ml	20
RSV	2 × 10^7^ TCID50/ml	20
*E. coli*	2 × 10^7^ cfu/ml	2.5

The concentration before irradiation and the dose for complete inactivation are indicated.

### Inactivation of eukaryotic cells

An important application of ionizing radiation in biotechnology is the treatment of mammalian cells, e.g. in the field of immune cell therapy. The inactivation of eukaryotic cells requires relatively low doses compared to microorganisms, due to the greater amount of DNA present in eukaryotes^33,34^. We irradiated the human natural killer cell line NK-92 in disposable bags with either 2.2, 6.6 or 11 Gy (Gray).

**Figure 5:**
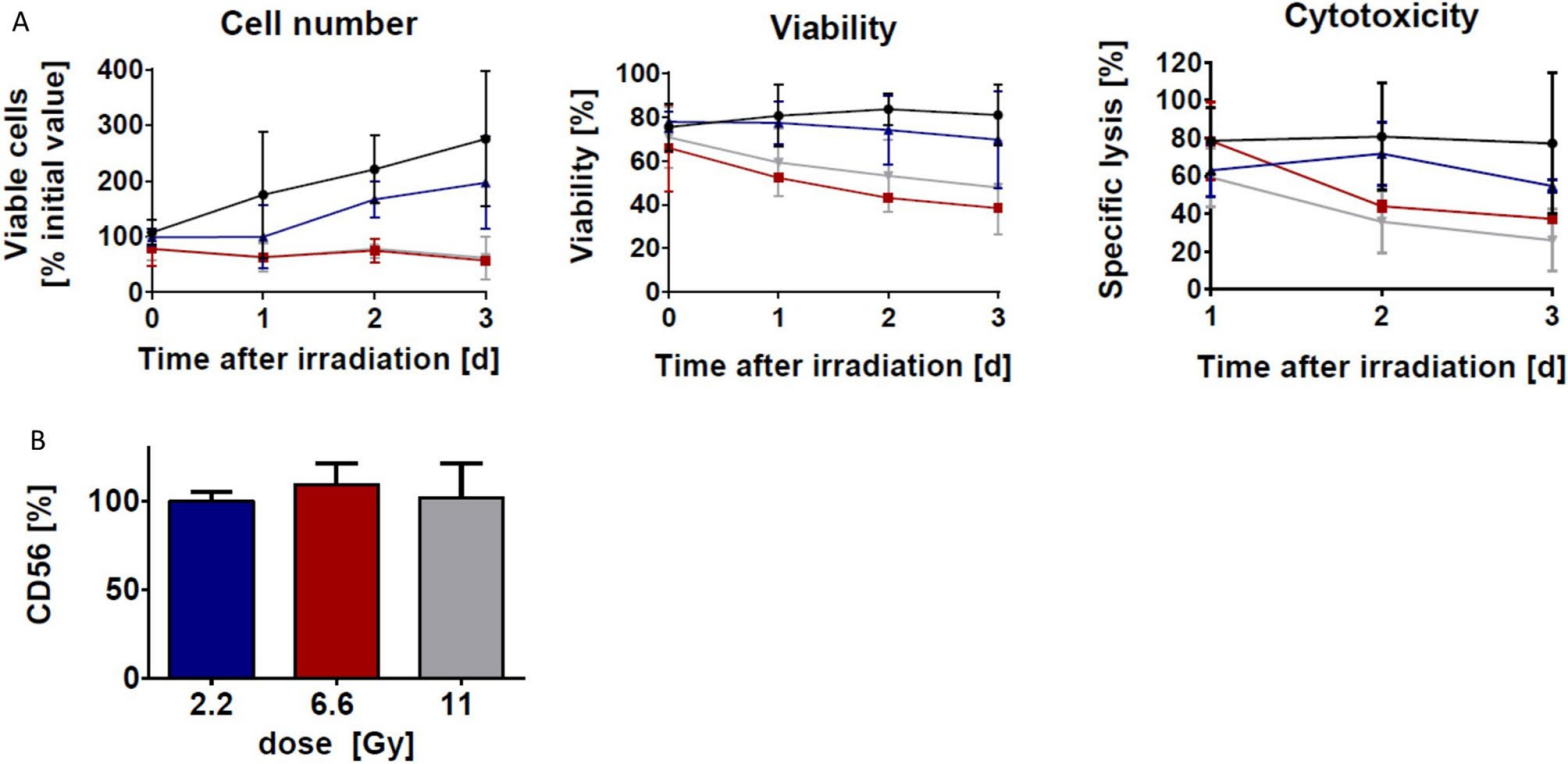
Electron beam irradiation causes a dose-dependent decrease of cell number, cell viability and cytotoxicity of NK-92 cells. (**A**) NK-92 cells were irradiated at 2.2 Gy (blue, n = 3), 6.6 Gy (red, n = 2) or 11 Gy grey, n = 4) and compared to non-irradiated control cells (black, n = 4) for growth, viability and activity against K562 cells (Effector:Target-ratio 5:1) during 3 days after irradiation. Values are displayed as mean ± standard deviation. (**B**) Surface expression of CD56 in NK-92 cells was analyzed by flow cytometry 2 days post irradiation. Mean fluorescence intensity (MFI) was compared to non-irradiated NK-92 cells. Mean values (n = 3) ± SD are shown, the non-irradiated control was set to 100%.

For the samples irradiated with 6.6 or 11 Gy, no proliferation was detected upon culturing the cells after the LEEI-treatment for up to 13 days (Fig. 5A, supplementary Fig. 3). On the other hand, the viability was still 38.4% (± 0.4%) and 47.8% (± 12.4%), respectively, three days after irradiation (Fig. 5A). For functional analysis the cells were incubated with the cancer cell line K562, and their cytotoxic activity was determined. When treated with 6.6 Gy and 11 Gy, respectively, the cells displayed specific lysis of 37.4% (± 1%) and 26.1% (± 9.5%) of the target cells three days after irradiation (Fig. 5A). The integrity of surface proteins was assessed by staining for the NK-specific marker CD56. No significant differences due to irradiation were detected when looking at the signal intensity via antibody staining (Fig. 5B).

### Generation of an inactivated RSV vaccine

**Figure 6:**
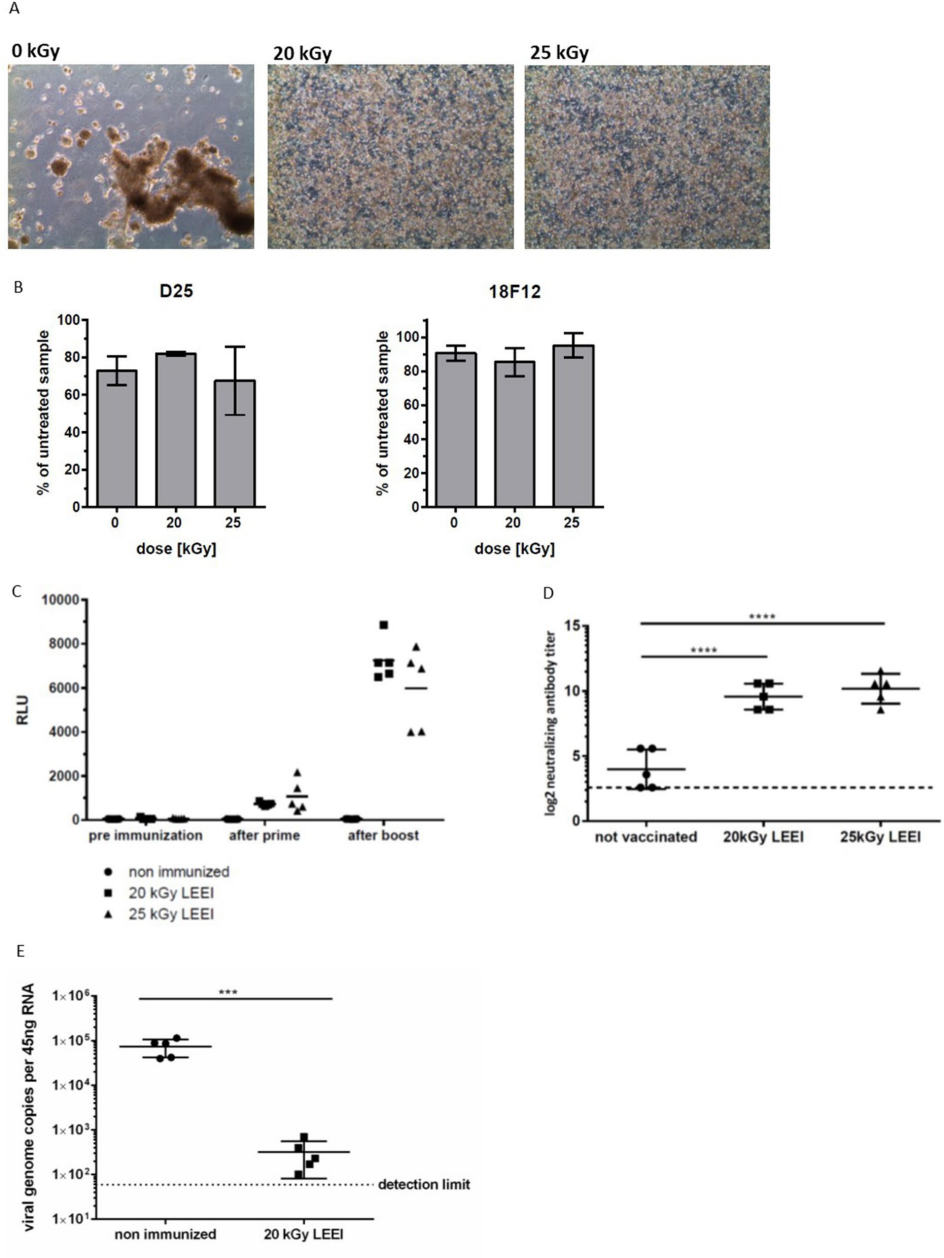
Inactivated RSV can be produced by LEEI and protects mice from infection. (**A**) Representative microscopic pictures of HEp2 cells at 5 days post infection with non-irradiated (0 kGy) RSV showing a cytopathic effect (CPE) and LEEI-inactivated (20 kGy and 25 kGy) RSV where no CPE is visible. (**B**) ELISA using two different antibodies targeting the RSV F surface protein of the virus (D25 n = 2; 18F12 n = 3). The signal intensity was normalized to the untreated sample which was set to 100%. Data are shown as mean ± SD. (**C**) RSV-specific binding antibodies in sera of mice immunized with inactivated RSV (20 kGy, rectangles and 25 kGy, triangles) or non-immunized animals as control (dots). Pre-immunization samples were taken one week before, prime blood samples three weeks after the first immunization and boost samples three weeks after the second immunization. Lines indicate the mean value of the analyzed group. (**D**) Virus neutralizing antibodies in sera of mice immunized with inactivated RSV (20 kGy, rectangles and 25 kGy, triangles) or non-immunized animals as control (dots). Prime sera were taken one week after the first immunization, boost sera one week after the second immunization. Dashed line represents the detection limit of the assay. Mean ± SD is indicated. (**E**) Viral load in lungs of immunized mice (RSV, inactivated with 20 kGy) and non-immunized mice 5 days after intranasal challenge with RSV. Viral load was determined with Real Time RT-PCR, the dashed line represents the detection limit of the assay. Mean ± SD is indicated.

To test whether the continuous system of LEEI would enable the production of inactivated vaccines, RSV was diluted to a titer of 2 × 10^7^ TCID50/ml and treated with LEEI of either 20 kGy or 25 kGy, based on previous experience^24^. Both doses inactivated the virus suspension (Fig. 6A). The conservation of antigens was investigated by analyzing the binding of two monoclonal antibodies to the F-protein, 18F12 and D25, respectively. The D25 antibody specifically detects the pre-fusion conformation of the protein, which has been linked to the induction of a potent protective immunity^35^. No significant differences were detected in the binding of both antibodies to the LEEI-treated RSV, indicating a high degree of conservation of the F-protein during the inactivation process (Fig. 6B).

The inactivated material was used to immunize mice in a prime-boost regimen. All animals developed RSV-specific antibodies, which strongly increased upon the booster immunization (Fig. 6C). Mice immunized with LEEI-treated RSV also displayed significantly higher amounts of virus neutralizing antibodies after boost compared to non-immunized animals (p < 0.0001) (Fig. 6D).

The animals immunized with the 20 kGy-irradiated RSV and the non-immunized controls were subjected to an intranasal RSV-challenge infection. Five days later, the viral load in the lungs was determined by Real-Time RT-PCR (Fig. 6E). Animals immunized with LEEI-inactivated RSV showed a statistically significant lower concentration of RSV in their lungs (p = 0.0008), which was 266-fold lower than in non-vaccinated controls. These data indicate that the automated process for LEEI of liquid solutions is suitable to generate inactivated viruses for vaccines.

## Discussion

We show that automated LEEI can be used to inactivate various organisms in liquid solution by delivering exact doses in a wide range, which renders the method suitable for numerous fields within biotechnology. Bacteria *(E. coli, B. cereus*) and viruses (Influenza A, RSV and Zika virus) were efficiently inactivated by the automated LEEI-processes, and the doses necessary were similar to those obtained with other ionizing radiation technologies, with viruses being generally more resistant than bacteria due to their lower content of nucleic acids^8^. Even *Bacillus*-spores, which are known to be highly resistant to radiation and other sterilization methods^36–38^, were readily inactivated by LEEI, although at much higher doses than *E. coli* cells. On the other hand, the antigenic material was well conserved and allowed recognition by antibodies or hemagglutination of erythrocytes. DNA-fragments generated by LEEI were still long enough to be detected in a diagnostic PCR for *B. cereus*. These findings are in line with data from other studies showing that ionizing radiation generates DNA-fragments of approx. 0.5–1 kb^39^. Therefore, liquids containing pathogens can be treated with LEEI and thereafter analyzed with standard diagnostic tools. This has important implications, e.g. for the analysis of potentially contagious patient samples containing highly pathogenic microbes which currently need be handled at a high containment level for the purposes of diagnostic analysis or need to be shipped to irradiation facilities^16^. Currently, chemical inactivation, often in combination with heat treatment, is commonly used for inactivating contagious pathogens in liquids. The addition of chemicals is time-consuming and complicates downstream assays due to chemical modification of nucleic acids and/or proteins in the sample^40^. Other procedures for sterilizing liquids e.g. blood products of human origin used in transfusion medicine usually involve ionizing radiation, mainly UV-irradiation. The major drawback is the requirement for photosensitizing chemicals for efficient inactivation with UV, due to the low penetration depth of the UV-irradiation^41^.

Another major advantage of LEEI compared to existing irradiation methods (gamma- and X-rays, high energy electrons) is the lack of considerable shielding requirements. Currently it is not possible to perform ionizing radiation for pathogen inactivation inside a standard biological laboratory. As a consequence, the material must be transported to specialized irradiation facilities, which makes the technology very complicated or even not feasible for many purposes.

In contrast, existing self-shielded LEEI-devices for surface treatment have a footprint of approx. 2 square-meters^42^, and the modules for generating the liquid film, described in this study, do not require substantially more space. Importantly, LEEI can be operated in any standard laboratory and the operating personal does not need additional radiation-protective clothing. Both principles for automated LEEI presented here are completely closed systems, guaranteeing personal and product protection. Electron irradiation is very accurate, as the beam current can be precisely controlled. This was evident in the reproducible dose curves when dosimetry was performed and in the killing curves of viruses and bacteria, using both the disposable bags and the continuous process. Another advantage of LEEI is its high dose rate, which enables fast processing. One bag containing the suspension was fully inactivated within less than 2 min. Similarly, in the continuous process, exposure time of the individual pathogens to the electron beam are below one second, which enables the inactivation of several liters per hour. In both cases, the dose does not have an influence on the processing duration. To achieve a similar inactivation with gamma-radiation (which has a much lower and decreasing dose rate based on the physical principles of its radioactivity and the decay of the material), the samples would need to be treated for up to several hours, depending on the pathogen^43,44^.

Besides delivering high doses to kill pathogens, automated LEEI also abolished proliferation of the human NK cell line NK-92, through the application of a much lower dose. The cell line is used in clinical trials for cancer therapy, and must be rendered proliferation-deficient before being applied to patients. This is currently performed through irradiation with 10 Gy^45^. At the same time, the cells need to maintain their cytotoxic anti-cancer cell activity over several days.

The NK-92 cells were unable to proliferate after LEEI with 6.6 and 11 Gy, but remained viable and cytotoxic towards cancer cells over at least three days. This indicates that LEEI could also be used in the generation of cell therapeutic strategies. Currently licensed cell therapies consist of autologous immune cells, and are therefore complex and expensive in manufacturing^46,47^. The usage of cell lines would have clear advantages, as these would enable off-the-shelf products, provided that the cells are proliferation-incompetent^45^. A detailed comparison of LEEI and the currently used gamma- or X-rays in immune cell irradiation still needs to be performed, but results obtained here show at least equal performances of LEEI-inactivated and state-of-the art irradiated NK-cells^30,48,49^.

The numerous studies demonstrating that ionizing irradiation is a promising technology for the generation of inactivated vaccines^8–10^ were confirmed by the LEEI-inactivated RSV. There is a particular demand for alternative inactivation methods for RSV, as the usage of formaldehyde has been associated with an enhanced course of the disease after subsequent natural infection^50^. In our study, RSV irradiated with the continuous process designed for multi-liter-applications still contained the RSV-F protein in the vaccine-critical prefusion conformation and was able to elicit specific binding and virus neutralizing antibodies. Moreover, immunized mice showed a significant (p = 0.0008) reduction in viral RNA in the lungs after challenge infection. In addition, Influenza A virus inactivated with automated LEEI showed a similar degree of antigen preservation as the material used in a previous study, where it was successfully used as a vaccine^23^.

Taken together, we present the first processes for automated LEEI of liquids, applicable in various fields of biotechnology. The developments enable inactivation of pathogens via ionizing radiation in a standard laboratory setting, including good manufacturing practice (GMP) and high biosafety levels. Critical structural components of viruses, bacteria and immune cells are maintained. The results form the basis for the development of irradiation-devices which can be placed into biological or medical laboratories and pharmaceutical production lines. The presented systems include possibilities for further upscaling, e.g. by using a longer roll in the continuous system, which would directly increase the throughput. The data underline the wide applicability of LEEI, ranging from the production of cell therapeutic strategies to inactivated material for diagnostic purposes, up to the generation of novel vaccines.

## Supplementary information

Supplementary file 1

## Data Availability

All data generated or analysed during this study are included in this published article (and its supplementary information files). The computer algorithm for measuring the liquid height is available in the supplementary material.
