# Publisher Correction: Automated application of low energy electron irradiation enables inactivation of pathogen- and cell-containing liquids in biomedical research and production facilities

**DOI:** 10.1038/s41598-020-73417-1

**Published:** 2020-10-23

**Authors:** Jasmin Fertey, Martin Thoma, Jana Beckmann, Lea Bayer, Julia Finkensieper, Susann Reißhauer, Beatrice Sarah Berneck, Leila Issmail, Jessy Schönfelder, Javier Portillo Casado, Andre Poremba, Frank-Holm Rögner, Bastian Standfest, Gustavo R. Makert, Lia Walcher, Ann-Kathrin Kistenmacher, Stephan Fricke, Thomas Grunwald, Sebastian Ulbert

**Affiliations:** 1 Fraunhofer Institute for Cell Therapy and Immunology IZI, Perlickstrasse 1, 04103 Leipzig, Germany; 2 Fraunhofer Institute for Manufacturing Engineering and Automation IPA, Nobelstrasse 12, 70569 Stuttgart, Germany; 3 Fraunhofer Institute for Organic Electronics, Electron Beam and Plasma Technology FEP, Winterbergstrasse 28, 01277 Dresden, Germany

Correction to: *Scientific Reports* https://doi.org/10.1038/s41598-020-69347-7, published online 30 July 2020

This Article contains a typographical error in the Introduction where,

“The inactivation of viruses and bacteria, however, requires high doses of rsubsequently inserted into the modadiation (in the kilogray range).”

should read:

“The inactivation of viruses and bacteria, however, requires high doses of radiation (in the kilogray range).”

