# Supplementary material for: Automated application of low energy electron irradiation enables inactivation of pathogen- and cell-containing liquids in biomedical research and production facilities
Source: Sci Rep. 2020 Jul 30;10:12786. doi: 10.1038/s41598-020-69347-7 (PMC7393095; doi:10.1038/s41598-020-69347-7)
Supplement: Supplementary file 1 [file 41598_2020_69347_MOESM1_ESM.pdf]

Supporting information

Automated application of low energy electron irradiation enables inactivation of pathogen- and cell - containing liquids in biomedical research and production facilities

Jasmin Fertey[1§], Martin Thoma[2§], Jana Beckmann[3], Lea Bayer[1], Julia Finkensieper[1], Susann Reißhauer[1], Beatrice Berneck[1], Leila Issmail[1], Jessy Schönfelder[3], Javier Portillo Casado [3], Andre Poremba[3], Frank-Holm Rögner[3], Bastian Standfest[2], Gustavo R. Makert[1], Lia Walcher[1], Ann-Kathrin Kistenmacher[1], Stephan Fricke[1], Thomas Grunwald[1#] and Sebastian Ulbert[1*]

[1] Fraunhofer Institute for Cell Therapy and Immunology IZI, Perlickstrasse 1, 04103 Leipzig, Germany

[2] Fraunhofer Institute for Manufacturing Engineering and Automation IPA, Nobelstrasse 12, 70569 Stuttgart, Germany

[3] Fraunhofer Institute for Organic Electronics, Electron Beam and Plasma Technology FEP, Winterbergstrasse 28, 01277 Dresden, Germany

[§] these authors contributed equally

# corresponding author referring to RSV: Thomas.grunwald@izi.fraunhofer.de

*corresponding author referring to LEEI: Sebastian.ulbert@izi.fraunhofer.de

Supplementary figures

Supplementary figure 1: LEEI device

Supplementary figure 2: laser interference measurement of the liquid height

Supplementary figure 3: LEEI stops proliferation of NK cells

Supplementary figure 4: detailed FACS measurement of CD56-positive NK cells

Supplementary tables

Supplementary table 1: measured volumes of different buffer compositions in the continuous irradiation module

Supplementary table 2: detailed cell numbers and normalized MFI calculations of three independent FACS measurements of CD56-positive NK cells

Supplementary note

Supplementary note 1: calculation of the liquid height

Supplementary Figure 1

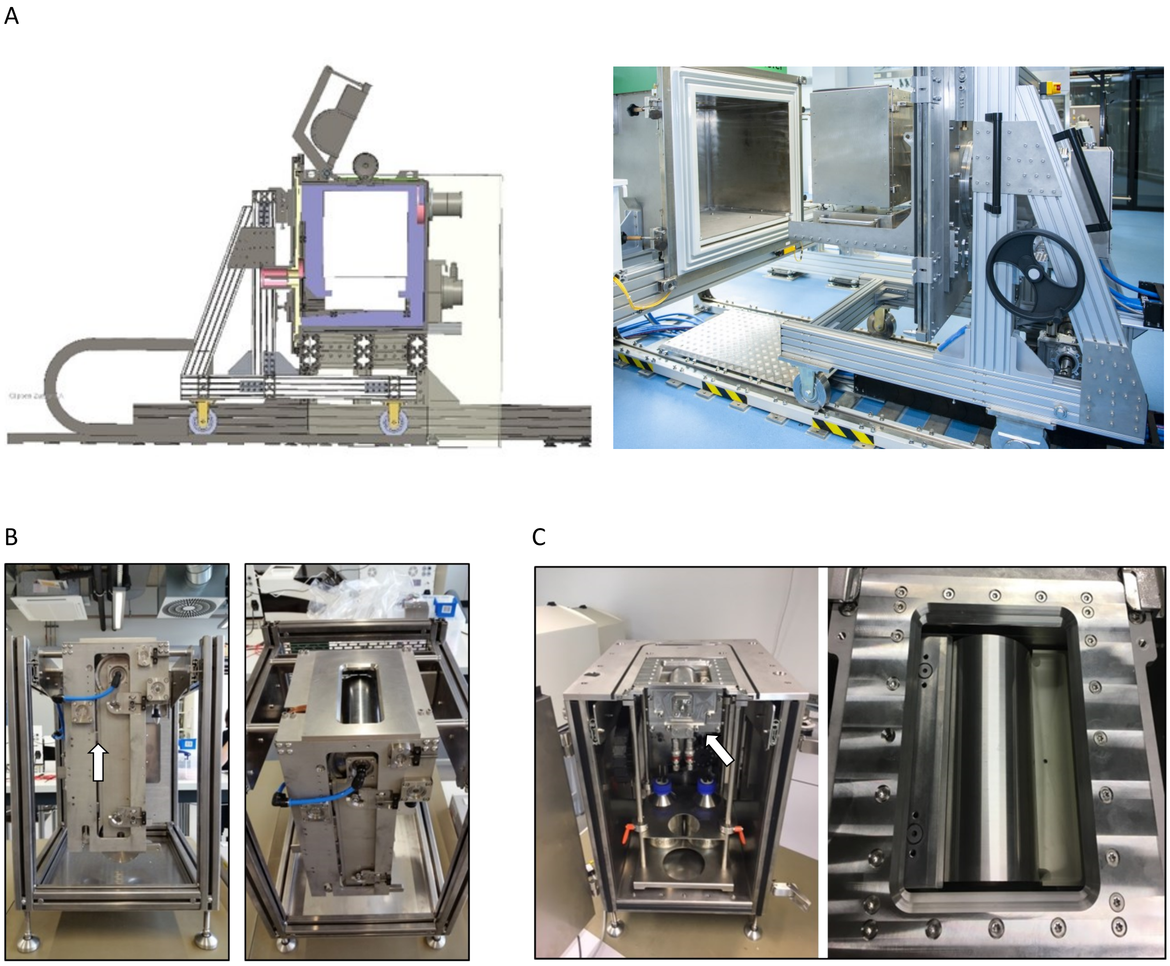

Supplementary figure 1: LEEI device

A: Schematic drawing (left) and photographic picture (right) of the electron beam facility containing the electron emitter (green, on top). The picture shows the opened irradiation chamber with the moveable recipient that carries one of the modules for liquid handling (closed metallic box). The irradiation chamber was designed as a research-scale prototype, so that different experimental modules for liquid handling can be inserted completely. This increases the overall footprint of the electron beam device.

B: Left: front view of the module for irradiating liquids in disposable bags. The bag is placed on the left side between the two conveyor belts (indicated by an arrow). Right: top view of the module where the bag is transported through the irradiation window for the electron beam.

C: Left: front view of the module for continuous processing. On the top, the reservoir for the liquid with the rotating roll is shown (indicated by an arrow). On the bottom, the bottles for the liquids (active and inactivated pathogen solution) are located. Right: top view of the module with the roll, that rotates in the reservoir and thereby transports the liquid through the irradiation window for the electron beam.

Supplementary Figure 2

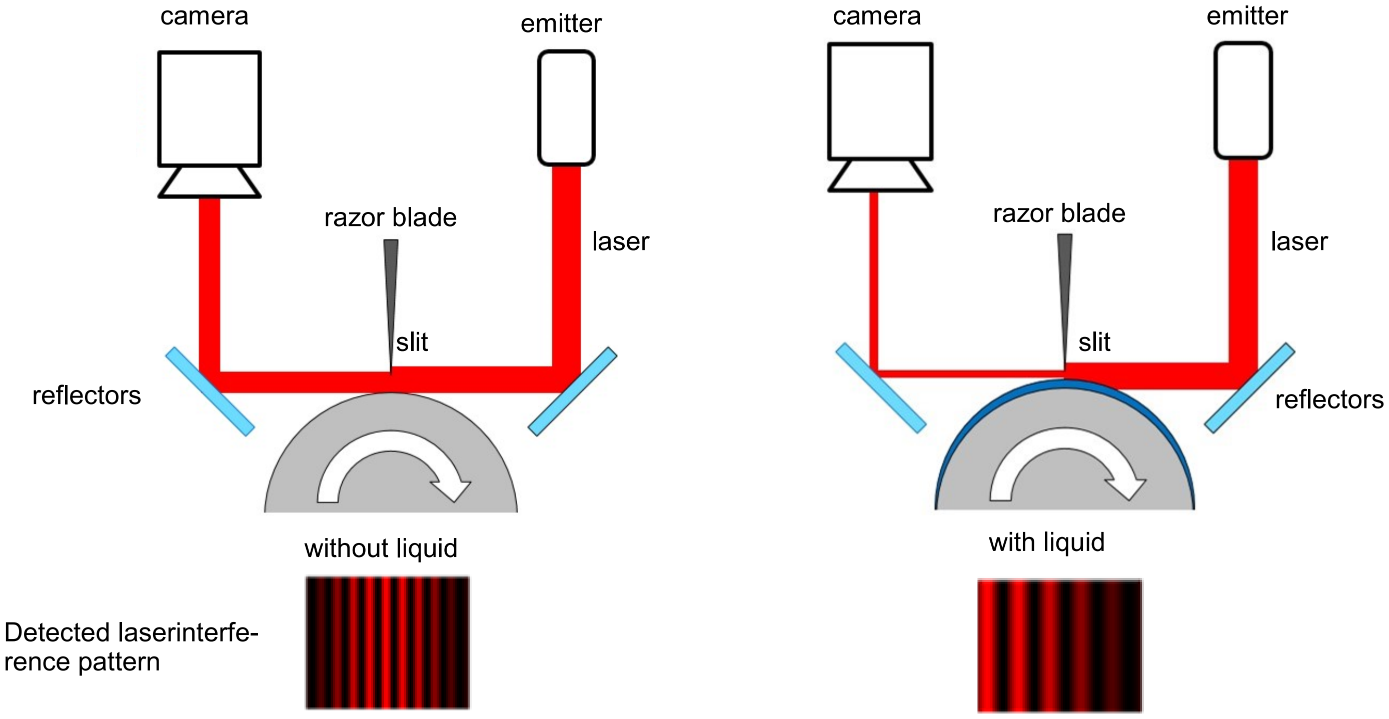

Supplementary figure 2: laser interference measurement of the liquid height.

Schematic drawing of the laser interference measurement setup. The emitter produces a laser beam, which is targeted through the measurement area to a camera (left). The installed razor blade on top of the measurement area creates a small slit. When liquid is processed (right), the width of the slit changes due to changes in the reflection and produces a different interference pattern than without liquid, which is detected by the camera. The measured slit change is subsequently translat-ed into the liquid´s height by a software (described in supplementary note 1).

Supplementary Figure 3

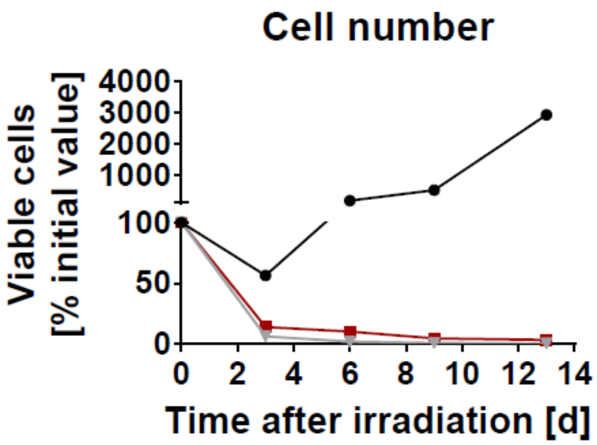

Supplementary figure 3: LEEI stops proliferation of NK cells

Long-term assessment of cell number of NK92 cells for 13 days after irradiation at 200 keV and 6.6 Gy (red, n = 1) or 11 Gy (grey, n = 1) and compared to non-irradiated control cells (black, n = 1). The experiment was carried out in technical triplicates and the mean values are indicated.

Supplementary Table 1

	Buffer + 20% Glycerol	Buffer + 10% Sucrose	cell culture super-natant
Mean Processed Volume [ml/min] (+/- SD)	60.9 (+/- 8.7)	43.9 (+/- 4.3)	27.9 (+/- 6.9)
Roll Speed [mm/s]	120	120	120

Supplementary table 1: measured volumes of different buffer compositions in the continuous irradiation module

Mean processed volumes in milliliters per minute of at least 8 independent experiments using different buffer compositions. Standard deviation is indicated in brackets. The circumferential speed of the roll was constantly at 120mm/s.

Supplementary Note 1

Calculation of the liquid height:

The calculation of the liquid height is based on the principle of diffraction at single slit and the resulting interference pattern on a screen. The distances of the intensity minima of the interference pattern is increased, the smaller the slit becomes. The liquid height h can be calculated using the wavelength λ of the laser, the distance α of the camera sensor to the slit and the distance d_k of the intensity minima as following:

$$h = \frac{\lambda * a}{d_k}$$

In the measurement setup, the interference pattern is digitized using a camera sensor. Hence, d_k can be obtained by multiplying the number of pixels and the pitch between two pixels ($PitchSize$).

$$h = \frac{\lambda * a}{Pixel * PitchSize}$$

The parameters for calculation are shown in the following table.

Designation	Parameter	Unit	Value
laser wavelength	λ	[nm]	685
distance camera – single slit	a	[mm]	210
distance of the minima in number of pixels	$Pixel$	-	-
pitch between two pixels	$PitchSize$	[µm]	3.45

Software:

The software for data processing was written in python (Python 3.7.4). After turning on the laser, the position of the razor blade has to be adjusted so that the interference image is projected on the camera chip. In the live image the user defines the area which will be used for measurement by choosing the image column and a width starting at this column.

Algorithm:

For each captured frame the area ,which was defined before is taken and averaged over the axis in parallel to lines of the interference pattern (2d array is reduced to an 1d array). This 1d array is appended to the result image as an additional column (before frame zero is an empty image).

The resulting image is filtered with a 2d Gaussian filter with user defined parameters for sigma-x and sigma-y. From the filtered image for each column (frame) the median of all distances between adjacent minima within the column is taken for the calculation of the slit height.

$$h = \frac{\lambda * a}{Pixel * PitchSize}$$

The calculated slit height for each frame is appended to the slit height plot. This plot can be filtered with an 1d Gaussian filter with user defined sigma-x.

Parameters used in this study:

Figure 1 B

height of area = 2000 pixel, width of area = 50 pixel, sigma-x = 4, sigma-y= 20, sigma-x height plot = 10

Figure 2 B
left: height of area = 2000 pixel, width of area = 50, sigma-x = 1, sigma-y= 20, sigma-x height plot = 10
right: height of area = 2000 pixel, width of area = 50, sigma-x = 1, sigma-y= 20, sigma-x height plot =10

Supplementary Figure 4

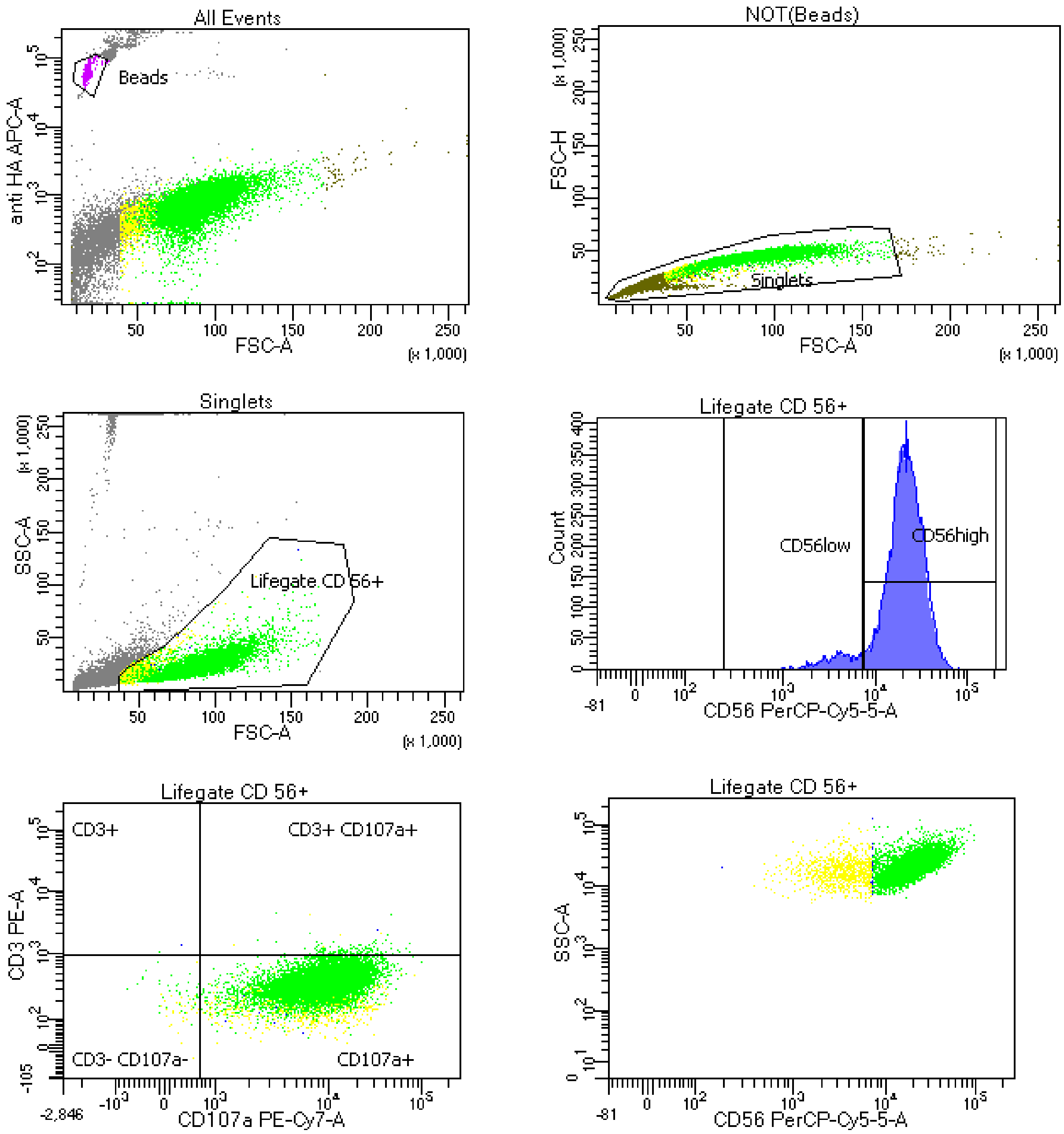

Supplementary figure 4: detailed FACS measurement of CD56-positive NK cells

To assess the expression of CD56 on cell surface , 2 µL of the monoclonal CD56 antibody (PerCP-Cy™5.5 mouse anti-human CD56 IgG1, κ, BD Biosciences, USA) were incubated at 4°C in the dark with 1 x 10^6 NK-92 cells for 20 min. For optimizing the fluorescence signal and possible spectral overlaps, compensation was performed with UltraComp™ eBeads (Thermo Fisher Scientific, USA). Precision Count Beads™ (BioLegend, USA) were pipetted into the stained NK-92 cells, prior to FACS measurement, to determine the absolute number of cells. Cell acquisitions were performed on a FACS Canto II flow cytometer (BD Biosciences, USA). The data was analysed using BD FACSDiva™ software (BD Biosciences, USA). Cells were identified using forward– and side scatter analysis. Gate for CD56+ (CD56high) was set based on fluorescence intensity in the indicated channel.

Supplementary Table 2

NK-92 cells

MFI CD 56	Counted CD56+cells	CD56 Signal [%]
	**NK92 non-irradiated**	
**17.07.2019**	16734	100
**24.07.2019**	15303	100
**21.08.2019**	22547	100
**mean**		**100,00**
	**200keV_2.2 Gy**	
**17.07.2019**	17397	103,96
**24.07.2019**	14455	94,46
**21.08.2019**	22925	101,68
**mean**		**100,03**
	**200keV_6.6 Gy**	
**17.07.2019**	20161	120,48
**24.07.2019**	14837	96,95
**21.08.2019**	24896	110,42
**mean**		**109,28**
	**200keV_11 Gy**	
**17.07.2019**	20418	122,02
**24.07.2019**	12677	82,84
**21.08.2019**	22634	100,39
**mean**		**101,75**

Supplementary table 2: detailed cell numbers and normalized MFI calculations of three independent FACS measurements of CD56-positive NK cells

Cells positive for CD56 were normalized by setting the non-irradiated control to 100%. MFI (mean fluorescence intensity) was calculated as described (Nowakowska et al. 2018).
